# P2X receptors as targets for the treatment of status epilepticus

**DOI:** 10.3389/fncel.2013.00237

**Published:** 2013-11-26

**Authors:** David C. Henshall, Miguel Diaz-Hernandez, M. Teresa Miras-Portugal, Tobias Engel

**Affiliations:** ^1^Department of Physiology and Medical Physics, Royal College of Surgeons in IrelandDublin, Ireland; ^2^Centre for the Study of Neurological Disorders, Royal College of Surgeons in IrelandDublin, Ireland; ^3^Department of Biochemistry and Molecular Biology IV, Veterinary School of Complutense, University of MadridMadrid, Spain; ^4^Institute of Investigación Sanitaria del Hospital Clinico San Carlos (IdISSC)Madrid, Spain

**Keywords:** anticonvulsant, ATP, epilepsy, hippocampus, interleukin-1β, microglia, neuroprotection

## Abstract

Prolonged seizures are amongst the most common neurological emergencies. Status epilepticus is a state of continuous seizures that is life-threatening and prompt termination of status epilepticus is critical to protect the brain from permanent damage. Frontline treatment comprises parenteral administration of anticonvulsants such as lorazepam that facilitate γ-amino butyric acid (GABA) transmission. Because status epilepticus can become refractory to anticonvulsants in a significant proportion of patients, drugs which act on different neurotransmitter systems may represent potential adjunctive treatments. P2X receptors are a class of ligand-gated ion channel activated by ATP that contributes to neuro- and glio-transmission. P2X receptors are expressed by both neurons and glia in various brain regions, including the hippocampus. Electrophysiology, pharmacology and genetic studies suggest certain P2X receptors are activated during pathologic brain activity. Expression of several members of the family including P2X_2_, P2X_4_, and P2X_7_ receptors has been reported to be altered in the hippocampus following status epilepticus. Recent studies have shown that ligands of the P2X_7_ receptor can have potent effects on seizure severity during status epilepticus and mice lacking this receptor display altered seizures in response to chemoconvulsants. Antagonists of the P2X_7_ receptor also modulate neuronal death, microglial responses and neuroinflammatory signaling. Recent work also found altered neuronal injury and inflammation after status epilepticus in mice lacking the P2X_4_ receptor. In summary, members of the P2X receptor family may serve important roles in the pathophysiology of status epilepticus and represent novel targets for seizure control and neuroprotection.

## Introduction

Status epilepticus is a potentially devastating neurological condition of continuous seizures. Current treatments are often unsuccessful in achieving complete seizure suppression, particularly when delivered late, so novel targets must be identified. ATP-gated ion channels—P2X receptors—are an interesting new focus of status epilepticus research. The pleiotropic effects of P2X receptor activation, including neuromodulation under conditions of excessive neuronal firing and indirect effects on excitability via control of neuroinflammation and gliosis offer a “multi-targeting” mode of action that may be particularly well suited to suppressing both the immediate pathologic brain activity and its downstream consequences. This review summarizes recent work on P2X receptors in status epilepticus, with particular emphasis on the P2X_7_ receptor (P2X_7_R), and speculates on the potential of these receptors as future drug targets for seizure control.

## Status epilepticus and limitations of current treatment

Status epilepticus is a state of continuous seizures, with an annual incidence ranging from 10 to 86 per 100,000 individuals (Chen and Wasterlain, 2006). Status epilepticus is traditionally defined as seizures lasting 30 min or more, but the current operational definition is clinical or electrographic seizures lasting beyond 5 min (Brophy et al., 2012). Status epilepticus represents a neurological emergency that is associated with profound morbidity and mortality. In humans and animal models, status epilepticus results in selective neuronal loss and gliosis, particularly within the hippocampus, as well as cognitive deficits and lasting hyper-excitability (Lowenstein, 2005; Chen and Wasterlain, 2006). Status epilepticus may result from metabolic disturbances, infection, drug toxicity or withdrawal, and non-compliance with the taking of anti-epileptic drugs (AEDs) (Brophy et al., 2012). Identifying the underlying cause of status epilepticus and treating it appropriately is paramount to alleviating the condition (Shorvon, 2011).

Pharmacological treatment of status epilepticus has been reviewed elsewhere (Lowenstein, 2005; Chen and Wasterlain, 2006) and new guidelines were recently published (Brophy et al., 2012). Initial therapy is to provide parenteral benzodiazepines such as lorazepam. Where benzodiazepines fail to control seizures, second-line therapy is usually with certain AEDs, including phenytoin. If both groups of drug fail and status epilepticus has become refractory, treatment options include ongoing intravenous combinations of the above or alternative treatments including hypothermia (Brophy et al., 2012). Recent work supports the use of the *N*-methyl-D-aspartate (NMDA) receptor antagonist ketamine for refractory status epilepticus (Synowiec et al., 2013).

## Animal models of status epilepticus

Animal models of status epilepticus have been critical for understanding the pathophysiology and treatment of status epilepticus. During status epilepticus there is a failure of the normal mechanisms for seizure termination, such as build-up of the anticonvulsant adenosine, acidosis or ion channel block. Other changes also accompany status epilepticus, including internalization of receptors for the inhibitory neurotransmitter γ-amino butyric acid (GABA) and externalization of receptors for the excitatory neurotransmitter glutamate (Wasterlain and Chen, 2006; Loscher, 2009). This is thought to underlie the development of benzodiazepine resistance which is common in status epilepticus. Optimal therapy is still lacking and there remains a need to identify other targets.

The most common animal models of status epilepticus use a chemoconvulsant or neurotoxin which is systemically administered or injected directly into the brain. Status epilepticus can also be triggered via electrical stimulation of the brain (e.g., perforant pathway, amygdala). Each has advantages and disadvantages, which have been reviewed elsewhere (Sperk, 1994; Loscher, 2002; Curia et al., 2008). To date, only the pilocarpine and kainic acid models have been used to investigate P2X modulation *in vivo*. Pilocarpine is a cholinergic agonist which produces status epilepticus and a pattern of hippocampal damage similar to that observed in epilepsy patients with mesial temporal sclerosis. However, induction of status epilepticus by pilocarpine appears to be secondary to peripheral immune responses and opening of the blood-brain barrier and the model is associated with high mortality, inter-animal variability in hippocampal pathology, and neuronal injury caused by ischemic as well as excitotoxic mechanisms (Fabene et al., 2007; Marchi et al., 2009). While the use of systemic kainic acid is also associated with variable hippocampal pathology, triggering status epilepticus using an intracerebral (e.g., intra-amygdala) injection of kainic acid produces a highly consistent focal and unilateral hippocampal lesion, with minimal mortality and reliable onset of spontaneous seizures (Li et al., 2008; Mouri et al., 2008; Liu et al., 2013). Such differences are important in critical evaluation of some of the conflicting findings with P2X ligands and genetically-modified mice.

## ATP-gated receptors; potential targets for seizure control?

ATP acts as a neurotransmitter/co-transmitter in the CNS where it has important neuromodulatory and trophic effects (Burnstock, 2008). ATP is released from neurons and glia in response to neuronal activity via exocytosis as well as through alternative routes, including hemichannels and other mechanisms (Lazarowski et al., 2003; Dale and Frenguelli, 2009). ATP can also accumulate because of release from damaged cells. Convulsive activity produces an overall reduction in brain ATP levels but intense activation of neuronal pathways also triggers ATP release (Dale and Frenguelli, 2009). Once released, ATP acts on ionotropic P2X and metabotropic P2Y receptors, and produces a mixture of excitatory and inhibitory effects [for review, see Burnstock (2007); Abbracchio et al. (2009)]. The other major class of purinoceptor, P1 receptors, is activated by adenosine. Adenosine is a potent anticonvulsant, and its important contribution to seizure control has recently been reviewed (Boison, 2013a,b).

P2X receptors are all ATP-gated ion channels, usually composed of heterotrimers of individual subunits encoded by seven different genes (*P2rx1-7*) that gate fast depolarizing sodium (Na^+^) and calcium (Ca^2+^) entry. A number of additional properties are attributed to P2X receptors. Extended activation of certain P2X receptors leads to the formation of a large pore with permeability to small molecules. This is best understood for the P2X_7_R, in which the response has been linked to a direct cytolytic effect, but other members of the P2X family may also form such channels (Surprenant et al., 1996; Di Virgilio et al., 1998; Virginio et al., 1999). The pore is not necessarily cytolytic, however, and there is controversy over whether the pore is instead formed by adjacent pannexin-1 channels (Duan et al., 2003; Pelegrin and Surprenant, 2006; Iglesias et al., 2008). The P2X_7_R has a number of other distinct characteristics. There is a large intracellular domain that enables it to directly interact with downstream pathways, including structural proteins (Kim et al., 2001). The receptor generally does not form heterotrimers and is found as a homotrimer, although recent work suggests it can interact with P2X_4_R in some cells (Craigie et al., 2013). The P2X_7_R also has low affinity for ATP, requiring mM levels for activation (Gever et al., 2006; Skaper et al., 2010). The implication is that the P2X_7_R is not activated under physiological conditions. The necessary conditions to generate sufficient extracellular ATP to activate P2X_7_R activation might include after cell lysis (e.g., neuronal necrosis due to excitotoxicity) or pathologic brain activity such as during prolonged or repeated brief seizures. Another feature of the P2X_7_R is that repeated agonist application under certain conditions results in sensitization and increased inward currents (Chessell et al., 1998; North and Surprenant, 2000; Armstrong et al., 2002).

Distribution of the P2X receptors has been previously reviewed, although characterization of their exact subunit composition in different tissues and cells is not yet complete (Norenberg and Illes, 2000; North and Surprenant, 2000; Gever et al., 2006). P2X receptors are found on neurons, where they may localize to both pre- and post-synaptic sites, and on non-neuronal cell types. The main subtypes expressed in the brain, including neurons in the hippocampus, are P2X2, 4 and 6 although P2X1, 3 and 5 receptor transcript and/or immunoreactivity has also been reported in the hippocampus (Papp et al., 2004; Dona et al., 2009; Engel et al., 2012a; Ulmann et al., 2013). The P2X_7_R was originally cloned from rat brain but considerable controversy has surrounded the exact localization in the brain. Early studies reported only microglial expression of the P2X_7_R in the adult brain (Collo et al., 1997). A number of groups have since reported P2X_7_R expression in neurons, including in the hippocampus (Deuchars et al., 2001; Armstrong et al., 2002; Dona et al., 2009; Engel et al., 2012a). However, the specificity of the antibodies used in those studies was questioned by the finding of widespread immunostaining with different antibodies in P2X_7_R knockout mice (Sim et al., 2004). Although that study concluded that the P2X_7_R was not expressed at appreciable levels in neurons of the hippocampus, other studies have identified the mRNA for P2X_7_R in neurons in the rodent hippocampus (Yu et al., 2008). New evidence for constitutive expression of the P2X_7_R in hippocampal neurons has come from studies of mice expressing enhanced green fluorescent protein (EGFP) under the control of the P2X_7_R promoter (Engel et al., 2012a,b; Jimenez-Pacheco et al., 2013). In the normal mouse brain, EGFP is seen in few dentate granule neurons and also in certain populations of neurons in the neocortex (Engel et al., 2012a,b; Jimenez-Pacheco et al., 2013).

The other major cell type expressing P2X_7_R in the brain is microglia (Collo et al., 1997; Rappold et al., 2006; Dona et al., 2009). Oligodendrocytes also express P2X_7_R (Matute et al., 2007; Yu et al., 2008). Although expression of P2X_7_R has been reported in cultured astrocytes (Duan et al., 2003), there is limited evidence for such expression *in vivo* (Yu et al., 2008; Engel et al., 2012a; Jimenez-Pacheco et al., 2013). Thus, multiple members of the P2X receptor family are expressed in brain where they may exert important modulatory effects on neuro- and glio-transmission (Khakh, 2001; Abbracchio et al., 2009).

## Expressional response of P2X receptors following status epilepticus

Injury to the brain produces widespread changes to the expression of P2X receptors (Burnstock, 2008). Following status epilepticus, there is a prominent increase in P2X_7_R immunoreactivity and functional responses in microglia (Rappold et al., 2006; Avignone et al., 2008). Protein levels of the P2X_7_R measured by immunoblotting also increase after status epilepticus in the hippocampus and neocortex, including in the synapto-dendritic compartment (Dona et al., 2009; Engel et al., 2012a; Jimenez-Pacheco et al., 2013). Transcript levels of P2X_7_R are increased in hippocampal neurons, particularly granule neurons, and microglia after status epilepticus (Avignone et al., 2008; Engel et al., 2012a). There has not been convincing *in vivo* evidence of changes to P2X_7_R expression in astrocytes or oligodendrocytes after status epilepticus (Rappold et al., 2006; Engel et al., 2012a; Jimenez-Pacheco et al., 2013).

There is less data on expressional responses of other P2X receptors after status epilepticus. Down-regulation of P2X_2_R has been reported after status epilepticus (Engel et al., 2012a) and P2X_2_R expression is also decreased in seizure-sensitive gerbils (Kang et al., 2003). For the P2X_4_R, studies have reported both up- and down-regulation in the hippocampus after status epilepticus (Avignone et al., 2008; Dona et al., 2009). The P2X_4_R was recently reported to be up-regulated on hippocampal microglia after status epilepticus in rats (Ulmann et al., 2013) but is expressed at lower levels in the seizure-sensitive gerbil (Kang et al., 2003). Hippocampal protein levels of P2X1, 3, and 5 receptors, as measured by immunoblotting, were all unchanged after status epilepticus (Engel et al., 2012a). Thus, status epilepticus produces select changes to levels of P2X receptors which are likely to result in altered responsiveness of glia and neurons to ATP signaling in the brain. A summary of status epilepticus-induced changes to P2X receptor expression is provided in Table 1.

**Table 1:** **P2X receptors in status epilepticus**.

	**Expression in seizure-relevant brain regions**	**Expressional response to status epilepticus**	**Effect of agonists/antagonists or knockout on status epilepticus, seizure-induced cell death and inflammation**
P2X_1_	Hippocampus^a^	*Hippocampus*	Not studied
	Cortex^b^	No change (W)^c^	
		Up-regulated (qPCR)^d^	
		*Cortex*	
		not studied	
P2X_2_	Hippocampus^e^^,^^f^	*Hippocampus*	Not studied
	Cortex^e^	Decreased (W)^c^	
		No change (W)^g^	
		*Cortex*	
		not studied	
P2X_3_	Hippocampus^h^	*Hippocampus*	Not studied
	Cortex^h^	No change (W)^3^	
		*Cortex*	
		not studied	
P2X_4_	Hippocampus^f^^,^^i^^,^^j^	*Hippocampus*	*P2X_4_ knock-out mice:*
	Cortex^i^^,^^j^	Increased (W, IH)^k^	Decreased seizure-induced cell death (i.p. KA)^k^
		No change (W)^c^^,^^g^	No effect on seizures (i.p. KA)^k^
		Up-regulated (qPCR)^d^	Decreased inflammation and microglia density (i.p. KA)^k^
		*Cortex*	No change in IL-1β levels (i.p. KA)^k^
		not studied	
P2X_5_	Hippocampus^l^	*Hippocampus:*	Not studied
	Cortex^l^	No change (W)^c^	
		*Cortex*	
		not studied	
P2X_6_	Hippocampus^f^^,^^i^	Not studied	Not studied
	Cortex ^i^		
P2X_7_	Hippocampus^c^^,^^m^^,^^n^	*Hippocampus:*	*Agonists (BzATP):*
	Cortex^n^^,^^o^	Increased (W, GFP)^c^^,^^g^	Increased seizures (i.a. KA)^c^
		Up-regulated (qPCR)^d^	No effect on seizures (Pilo)^q^
		Increased (IH)^p^	Increased microglia activation (Pilo)^r^
		*Cortex:*	Increase in astrocyte loss (Pilo)^s^
		Increased (W, GFP)^o^	Increased TNF-α immunoreactivity (Pilo)^t^
			Decreased seizure-induced cell death (Pilo)^t^
			*P2X_7_R knock-out mouse:*
			Decreased seizures (i.a. KA)^c^
			Increased seizures (Pilo)^q^
			No effect on seizures (i.p. KA and i.p. Pic)^q^
			*Antagonists (A-43, A-74, BBG, OxATP, IgG-P2X_7_):*
			Decreased seizures (i.a. KA)^c^^,^^o^
			Increased seizures (Pilo)^q^
			Decreased seizure-induced cell death (i.a. KA)^c^
			Increased seizure-induced cell death (Pilo)^t^
			Decreased astrocyte loss (Pilo)^s^
			Decreased microglia activation (i.a. KA and Pilo)^c^^,^^r^
			Decreased Il-1β levels (i.a. KA)^c^

*A-43, A-438079; A-74, A-740003; BzATP, 2′,3′-O-(4-benzoylbenzoyl)-adenosine 5′-triphosphate; OxATP, oxidised ATP GFP, P2X_7_R-GFP-reporter mouse; i.a. KA., intra-amygdala KA; i.p. KA, intra-peritoneal KA; Pilo, Pilocarpine; i.p. Pic, intra-peritoneal Picrotoxin; IgG-P2X_7_, anti-P2X_7_ antibodies; Il-1β, interleukin-1beta; qPCR, quantitative polymerase chain reaction; IH, Immunohistochemistry; TNF-α, Tumor necrosis factor alpha; W, Western blot*.

a Cavaliere et al., 2007;

b Lalo et al., 2008;

c Engel et al., 2012a;

d Avignone et al., 2008;

e Kanjhan et al., 1999;

f Rubio and Soto, 2001;

g Dona et al., 2009;

h Seguela et al., 1996;

i Collo et al., 1996;

j Le et al., 1998;

k Ulmann et al., 2013;

l Guo et al., 2008;

m Armstrong et al., 2002;

n Yu et al., 2008;

o Jimenez-Pacheco et al., 2013;

p Rappold et al., 2006;

q Kim and Kang, 2011;

r Choi et al., 2012;

s Kim et al., 2011a;

t *Kim et al., 2011b*.

## Role of P2X receptors in brain excitability

Under physiological circumstances, P2X gated currents at synapses are thought to be small and not uniformly detected (North, 2002; Khakh and North, 2006). Intracellular recordings have estimated the ATP-dependent fast excitatory component to comprise 5–20% of the total synaptic current in CA1 pyramidal cells (Pankratov et al., 2002). The real significance of P2X receptor-mediated current may be to facilitate Ca^2+^ entry into cells. Together with the presynaptic location of certain P2X receptors, this implicates them in control of neurotransmitter release (Sperlagh et al., 2007). The distribution of P2X receptors at synapses—particularly at the periphery of the post-synaptic density—suggests their contribution becomes more important under conditions of intense neuronal activity (Khakh and North, 2006).

A number of studies have investigated the effects of P2X receptor activation or blockade on hippocampal excitability and there is evidence for both pro- and anti-excitatory consequences. An excitatory effect of the P2X agonist α, β-meATP was found in rat hippocampal slices (Ross et al., 1998) and hippocampal slices from seizure-prone mice release more extracellular ATP when stimulated (Wieraszko and Seyfried, 1989). In contrast, blockade of post-synaptic P2X receptors was observed to facilitate long-term potentiation, suggesting some P2X receptor functions restrict certain aspects of synaptic plasticity (Pankratov et al., 2002). P2X_2_R are present on the presynaptic terminals of CA3 axons (Schaffer collaterals) that terminate on inhibitory interneurons in the CA1 subfield and are thought to function as a physiological brake on excessive neuronal activity (Khakh and North, 2006). Activation of P2X_2_R enhances release of excitatory neurotransmitter onto CA1 interneurons, which in turn increases release of inhibitory neurotransmitter to reduce excitatory drive onto CA1 pyramidal neurons (Khakh et al., 2003). Notably, down-regulation of the P2X_2_R has been reported in models of status epilepticus, suggesting loss of this receptor might represent a novel “channelopathy” (Engel et al., 2012a).

P2X_7_R also mediate effects on hippocampal excitability. Stimulation of P2X_7_R present on the presynaptic terminals of mossy fibers reduced excitatory field potentials recorded in the CA3 subfield (Armstrong et al., 2002). These data are consistent with a model whereby pre-synaptic P2X_7_R are activated during high-level neuronal excitability and function to reduce further release of glutamate from mossy fiber terminals (Armstrong et al., 2002). The P2X_7_R effect to decrease transmitter release probability at mossy fiber synapses is therefore the opposite of what was found for pre-synaptically-located P2X_2_R, which enhanced transmitter release probability, but functionally these actions are synergistic, to limit over-excitation within the hippocampus. These results are also consistent with the main effects of ATP being pre-synaptic, not post-synaptic in the hippocampus (Khakh et al., 2003). Other recent work in a model of recurrent epileptiform activity found a small effect of P2X_7_R antagonists against slow field potentials induced by potassium-bicuculline treatment of rat cortical slices (Klaft et al., 2012).

Overall, the properties of the P2X system—activation under high levels of neuronal activity—are particularly relevant to status epilepticus and raise the prospect of a class of receptor that when targeted may influence pathologic brain activity while leaving normal neurotransmission largely unaffected. Nevertheless, until very recently, no study had directly investigated the effect of ligands acting at P2X receptors on status epilepticus.

## *In vivo* studies with P2X_7_R ligands in status epilepticus

There has been significant interest in P2X_7_R ligands as therapeutics for neurological conditions (Skaper et al., 2010). The leading clinical application of P2X_7_R receptor antagonists is for treatment of neuropathic pain but there are indications in acute neurologic injuries. For example, P2X_7_R antagonists have been reported to reduce injury or inflammation following intracerebral hemorrhage (Chen et al., 2013) and global ischemia (Yu et al., 2013). In focal cerebral ischemia, protective effects were reported in some studies (Melani et al., 2006; Arbeloa et al., 2012) but not others (Le Feuvre et al., 2003). Also of interest, P2X_7_R agonists have been shown to trigger a protective state, a form of “chemical preconditioning,” that rendered cardiac tissue resistant to subsequent ischemia (Vessey et al., 2011). Protective effects have also been reported for P2X_7_R antagonists in models of neurodegeneration, including Huntington's disease (Diaz-Hernandez et al., 2009), Parkinson's disease (Marcellino et al., 2010), amyotrophic lateral sclerosis (Cervetto et al., 2013) and Alzheimer's disease (Diaz-Hernandez et al., 2012; Murphy et al., 2012).

P2X_7_R antagonists have been reported to produce potent anticonvulsant effects in some, but not all models of status epilepticus (see Table 1). Studies by the authors demonstrated that a central (intracerebroventricular) injection the P2X_7_R antagonists BBG or A-438079 resulted in as much as a 50% reduction in electrographic seizures during status epilepticus triggered by intra-amygdala microinjection of kainic acid in mice (Engel et al., 2012a; Jimenez-Pacheco et al., 2013). Behavioral convulsions were also reported to be reduced in mice treated with A-438079 prior to status epilepticus (Jimenez-Pacheco et al., 2013). Experiments using the “Pfizer” P2X_7_R knockout mice (Solle et al., 2001) supported these pharmacological studies, with seizure severity reduced compared to wild-type animals. Further complementing these findings, intracerebroventricular injection of a P2X_7_R blocking antibody suppressed seizures while BzATP, a P2X_7_R agonist, exacerbated seizures in the model (Engel et al., 2012a). Analysis of the hippocampus and neocortex of mice pre-treated with P2X_7_R antagonists found reductions in neuronal death, microgliosis and interleukin-1β (Engel et al., 2012a; Jimenez-Pacheco et al., 2013). Treatment of mice with P2X_7_R antagonists 20 min after triggering status epilepticus - a more clinically-relevant scenario—also reduced seizure severity and protected the hippocampus (Engel et al., 2012a). Finally, injection of A-438079 1 h after status epilepticus began, at a time when sensitivity to lorazepam was reduced, also had modest seizure-suppressive effects (Engel et al., 2012a). This finding is important since it supports the possible use of P2X ligands as adjunctive treatments for status epilepticus alongside frontline drugs such as lorazepam.

These findings add complexity to the pathophysiological functions assigned to the P2X_7_R in the brain. *In vitro* data had supported the P2X_7_R as a “physiological brake” on overexcitation (Armstrong et al., 2002) but these *in vivo* data indicate blocking the P2X_7_R reduces hyper-excitation. It will be important to establish mechanisms that account for the observed *in vivo* effects of P2X_7_R antagonists against status epilepticus. Direct effects of P2X_7_R on neuronal activity may behave differently *in vivo* during status epilepticus. For example, the pre-synaptic P2X_7_R thought present on mossy fibers facilitating rather than opposing glutamate release, as demonstrated for P2X_2_R (Khakh et al., 2003). Also, perhaps blocking P2X_7_R on glia (e.g., microglia) confers anti-excitatory effects that functionally supersede pre-synaptic effects limiting transmitter release. As always, findings based mainly on pharmacology require careful consideration of the specificity of the ligands involved (Anderson and Nedergaard, 2006).

## Other P2X receptor ligands in status epilepticus

The only other member of the P2X family for which *in vivo* data exist in a model of status epilepticus is the P2X_4_R. Mice lacking the P2X_4_R display a reduction in neuronal death after status epilepticus, although seizures themselves were not altered in these mice (Ulmann et al., 2013). Notably, while some inflammatory signaling was also reduced, the induction of interleukin-1β was not found to be different, supporting other work linking modulation of this pathway to the P2X_7_R (see below).

We can speculate that targeting other members of the P2X family would have seizure-modulating effects *in vivo*, although there are fewer ligands selective for the other P2X receptors. An obvious candidate would be an agonist of the P2X_2_R. Activation of this receptor promotes inhibitory transmission within the hippocampus (Khakh and North, 2006). Delivery of such a ligand might enhance endogenous mechanisms of seizure suppression.

## Glia-related functions of the P2X_7_R in status epilepticus

The immediacy of the seizure-suppressive effects of P2X_7_R antagonists implies a direct action on neurons, for which there is supporting evidence (Armstrong et al., 2002; Engel et al., 2012a,b). However, expression and activation of the P2X_7_R on glia may have important effects on excitability that influence the pathophysiology and outcome of status epilepticus. First, P2X_7_R activation has been associated with production of cytokines from microglia (Ferrari et al., 1996; Chakfe et al., 2002). In particular, activation of the P2X_7_R leads to processing and release of interleukin-1β, which is a potent pro-convulsive molecule implicated as a target for seizure control (Vezzani et al., 2010). Activated microglia also exacerbated excitotoxicity in hippocampal cultures, an effect shown to be P2X_7_R-dependent (Bernardino et al., 2008). Thus, targeting P2X_7_R effects on microglia may reduce post-status epilepticus inflammation and susceptibility to excitotoxicity.

P2X_7_R have been implicated in certain trophic functions, including the activation and proliferation of microglia. This serves both restorative and pathologic functions after status epilepticus, contributing to tissue repair but also releasing pro-inflammatory mediators which may contribute to hyper-excitability (Devinsky et al., 2013). Increased P2X_7_R expression and receptor activation was found to transform resting microglia to an activated state (Monif et al., 2009). Activation of the pore-forming function of the P2X_7_R was also found to be required for microglial proliferation (Monif et al., 2009). Consistent with this model, blockade of P2X_7_R reduces microglia activation after status epilepticus (Kim et al., 2009; Choi et al., 2012; Engel et al., 2012a). Targeting the pore-forming functions of the P2X_7_R may therefore be a novel approach to limit microglia responses following status epilepticus.

Astrocytes represent another non-excitable cell involved in mediating the effects of ATP. There is *in vitro* evidence that P2X_7_R activation on astrocytes triggers glutamate release, perhaps directly through the channel/pore (Duan et al., 2003). Such P2X_7_R-mediated glutamate release from astrocytes may contribute to astrocyte signaling, promote excitability or even excitotoxicity. P2X_7_R activation on astrocytes may also serve a cell-killing function. Injection of rats with the P2X_7_R agonist BzATP was found to reduce astrocyte numbers after status epilepticus and P2X_7_R antagonists prevented astrocyte death (Kim et al., 2009, 2011a). Thus, secondary effects of P2X7R ligands will need to be considered as modulation of astrocyte number or activation has profound effects on excitability in the brain (Boison, 2008). Oligodendrocytes are also sensitive to the toxic effects of ATP acting on P2X_7_R (Matute et al., 2007).

Finally, it has emerged that the P2X_7_R may promote axonal growth and branching in the hippocampus (Diaz-Hernandez et al., 2008). Studies have also reported that P2X_7_R antagonists improved recovery after spinal cord injury (Peng et al., 2009). Synaptic reorganization is long-recognized following status epilepticus and has been implicated in establishing recurrent excitatory circuits (e.g., mossy fiber sprouting) that may contribute to epileptogenesis or chronic epilepsy (Houser et al., 2012). The benefits from targeting the P2X_7_R could therefore extend well beyond the initial period of seizure activity. As with effects on astrocytes and microglia, these data suggest targeting the P2X_7_R may have pleiotropic effects and the timing of manipulations or site of targeting may be critical to obtain optimal therapeutic benefit.

**Figure 1:**
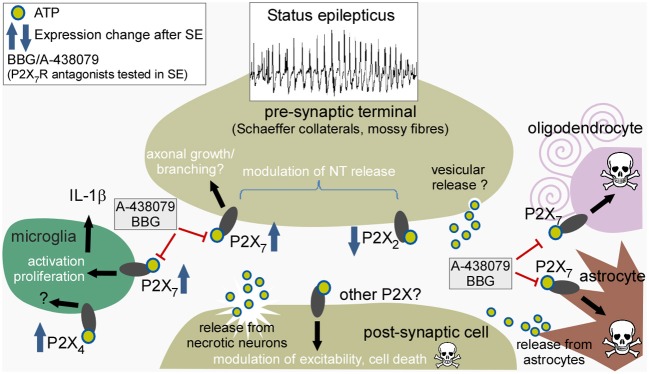
**Potential sites of action of ATP released during status epilepticus, expressional responses of individual P2X receptors, and consequences of receptor activation**. Cartoon depicts the various different cell types reported to express P2X receptors and their presumed cellular locations. ATP is released during sustained neuronal activity and from damaged neurons to act pre- and post-synaptically on neurons, particularly targeting pre-synaptic receptors to modulate neurotransmitter release. ATP may also act on receptors of microglia to promote activation and release of interleukin-1β, and act on astrocytes and oligodendrocytes to trigger cell death. Drugs such as A-438079 and BBG have been reported to reduce seizures and gliosis after status epilepticus. SE, status epilepticus; IL-1β, interleukin-1β.

Figure 1 summarizes the mechanisms of ATP release during seizures, the receptors upon which ATP may act, expressional changes, and some of the downstream effects of P2X receptor modulation relevant to the pathophysiology of status epilepticus.

## Limitations of targeting P2X_7_ receptors

While there is significant support for a role for P2X_7_R in neuronal injury and/or glial activation, there are also conflicting findings. Excitotoxic injury has been reported to be unchanged in mice lacking P2X_7_R or in response to P2X_7_R antagonists (Le Feuvre et al., 2003). Recent work by Frenguelli's group found a limited role for P2X receptors in electrically-evoked seizure-like activity in rat hippocampal slices, and no effect of P2X_7_R antagonists on these events (Lopatar et al., 2011). Similarly, no effects of P2X_7_R antagonists on *in vitro* epileptiform activity were detected in acute cortical slices from epileptic rats (Klaft et al., 2012). Inter-species differences could be to blame and have been reported for the P2X_7_R (Chessell et al., 1998). Last, Kang's group reported that pilocarpine-induced seizures were in fact exacerbated in mice lacking P2X_7_R and in wild-type animals treated with P2X_7_R antagonists (Kim and Kang, 2011). These data are in sharp contrast to the findings with intra-amygdala kainic acid-induced status epilepticus (Engel et al., 2012a; Jimenez-Pacheco et al., 2013). Although the reasons underlying these contradictory findings are uncertain they may relate to the specificity and dose of the ligands used, models or genetic tools. Indeed, the findings may be peculiar to the pilocarpine model because seizures induced by kainate or picrotoxin were not exacerbated in mice lacking P2X_7_R in that study (Kim and Kang, 2011). Another factor may be the variable influence of tissue responses affecting the pharmacological properties of the receptor. The P2X_7_R is inhibited by acidosis (North, 2002), which develops during status epilepticus (Ziemann et al., 2008). Does P2X_7_R blockade develop regardless of pharmacological antagonism in some models of status epilepticus? Further studies will be needed to resolve these complicated issues.

## Other considerations in the development of P2X ligands for status epilepticus

New P2X receptor ligands have recently emerged while several of the known P2X antagonists show potentially suitable drug profiles. This includes the P2X_7_R antagonist A-438079, a relatively small molecule that crosses the blood-brain barrier following systemic delivery (McGaraughty et al., 2007). A recently developed P2X_7_R antagonist is also able to pass the blood brain barrier (Bhattacharya et al., 2013). More complete reviews on the potential of P2X receptor ligands as drugs can be found elsewhere (Burnstock, 2008; North and Jarvis, 2013).

Despite the need for new therapies, there has been disengagement of several of the major pharmaceutical companies from developing new anticonvulsants and treatments for epilepsy. It has been suggested that the identification of additional clinical applications (i.e., non-epileptic conditions) would make a potential anticonvulsant drug candidate significantly more attractive for development (Bialer and White, 2010). One example is with the effectiveness of certain AEDs for the treatment of pain. Notably, a key area of deployment of P2X_7_R antagonists is for the treatment of pain conditions (Trang et al., 2012; Alves et al., 2013).

## Remaining challenges

There are opportunities for the use of P2X receptor ligands in the control or prevention of seizures but significant challenges remain. Foremost, P2X_7_R antagonists need to be evaluated in other models because of the conflicting reports between kainic acid and pilocarpine models. These issues are not unique to status epilepticus; conflicting data on the P2X_7_R have emerged in the stroke field (Le Feuvre et al., 2003; Melani et al., 2006; Yanagisawa et al., 2008; Arbeloa et al., 2012). A genetic approach will be needed to confirm specific drug effects are lost in animals lacking the P2X_7_R, although such studies are not without problems (Nicke et al., 2009; Masin et al., 2012).

Assuming P2X_7_R antagonists display consistent anticonvulsive profiles, work will be needed to explore dosing and route of delivery. Do these drugs suppress seizures when given systemically and are there off-target effects? Increased attention is needed on assessing the pleiotropic actions of these drugs, and consideration given to looking for effects on glia and inflammation, including long after the initial period of status epilepticus. Are anti-inflammatory effects of P2X_7_R antagonists countered by trophic effects that promote gliosis? P2X ligands may have effects against spontaneous seizures, which would raise their potential as future AEDs. P2X receptors serve important roles in neurodevelopment and ligands may also have potential to treat seizures in the developing brain, a condition currently poorly served by available treatments (Slaughter et al., 2013). It will also be important to determine the mechanism(s) controlling P2X receptor expression after status epilepticus. Indeed, efforts to “rescue” the post-status epilepticus decline in P2X_2_R levels could promote inhibitory transmission. Last, we lack relevant human data. Although there is evidence for altered expression of P2X receptors in human epilepsy (Jimenez-Pacheco et al., 2013), their expression and function in status epilepticus is unknown.

In summary, there is increasing evidence for a role for ATP and P2X receptors in seizure states such as status epilepticus and epilepsy. Certain P2X receptors may represent novel drug targets for seizure control. Targeting these receptors could provide frontline or adjunctive seizure suppression during status epilepticus, as well as influencing post-injury glial function that may help mitigate outcomes including epileptogenesis.

### Conflict of interest statement

The authors declare that the research was conducted in the absence of any commercial or financial relationships that could be construed as a potential conflict of interest.

## References

[B1] Abbracchio M. P. Burnstock G. Verkhratsky A. Zimmermann H. (2009). Purinergic signalling in the nervous system: an overview. Trends Neurosci. 32, 19–29 10.1016/j.tins.2008.10.001 19008000

[B2] Alves L. A. Bezerra R. J. Faria R. X. Ferreira L. G. Da Silva Frutuoso V. (2013). Physiological roles and potential therapeutic applications of the P2X7 receptor in inflammation and pain. Molecules 18, 10953–10972 10.3390/molecules180910953 24013409 PMC6270334

[B3] Anderson C. M. Nedergaard M. (2006). Emerging challenges of assigning P2X7 receptor function and immunoreactivity in neurons. Trends Neurosci. 29, 257–262 10.1016/j.tins.2006.03.003 16564580

[B4] Arbeloa J. Perez-Samartin A. Gottlieb M. Matute C. (2012). P2X7 receptor blockade prevents ATP excitotoxicity in neurons and reduces brain damage after ischemia. Neurobiol. Dis. 45, 954–961 10.1016/j.nbd.2011.12.014 22186422

[B5] Armstrong J. N. Brust T. B. Lewis R. G. Macvicar B. A. (2002). Activation of presynaptic P2X7-like receptors depresses mossy fiber-CA3 synaptic transmission through p38 mitogen-activated protein kinase. J. Neurosci. 22, 5938–5945 12122056 10.1523/JNEUROSCI.22-14-05938.2002 PMC6757920

[B6] Avignone E. Ulmann L. Levavasseur F. Rassendren F. Audinat E. (2008). Status epilepticus induces a particular microglial activation state characterized by enhanced purinergic signaling. J. Neurosci. 28, 9133–9144 10.1523/JNEUROSCI.1820-08.2008 18784294 PMC6670931

[B7] Bernardino L. Balosso S. Ravizza T. Marchi N. Ku G. Randle J. C. (2008). Inflammatory events in hippocampal slice cultures prime neuronal susceptibility to excitotoxic injury: a crucial role of P2X7 receptor-mediated IL-1beta release. J. Neurochem. 106, 271–280 10.1111/j.1471-4159.2008.05387.x 18384650

[B8] Bhattacharya A. Wang Q. Ao H. Shoblock J. R. Lord B. Aluisio L. (2013). Pharmacological characterization of a novel centrally permeable P2X7 receptor antagonist: JNJ-47965567. Br. J. Pharmacol. 170, 624–640 10.1111/bph.12314 23889535 PMC3792000

[B9] Bialer M. White H. S. (2010). Key factors in the discovery and development of new antiepileptic drugs. Nat. Rev. Drug Discov. 9, 68–82 10.1038/nrd2997 20043029

[B10] Boison D. (2008). The adenosine kinase hypothesis of epileptogenesis. Prog. Neurobiol. 84, 249–262 10.1016/j.pneurobio.2007.12.002 18249058 PMC2278041

[B11] Boison D. (2013a). Adenosine kinase: exploitation for therapeutic gain. Pharmacol. Rev. 65, 906–943 10.1124/pr.112.006361 23592612 PMC3698936

[B12] Boison D. (2013b). Role of adenosine in status epilepticus: a potential new target? Epilepsia 54(Suppl. 6), 20–22 10.1111/epi.12268 24001064 PMC3767194

[B13] Brophy G. M. Bell R. Claassen J. Alldredge B. Bleck T. P. Glauser T. (2012). Guidelines for the evaluation and management of status epilepticus. Neurocrit. Care 17, 3–23 10.1007/s12028-012-9695-z 22528274

[B14] Burnstock G. (2007). Physiology and pathophysiology of purinergic neurotransmission. Physiol. Rev. 87, 659–797 10.1152/physrev.00043.2006 17429044

[B15] Burnstock G. (2008). Purinergic signalling and disorders of the central nervous system. Nat. Rev. Drug Discov. 7, 575–590 10.1038/nrd2605 18591979

[B15a] Cavaliere F. Amadio S. Dinkel K. Reymann K.G. Volonte C. (2007). P2 receptor antagonist trinitrophenyl-adenosine-triphosphate protects hippocampus from oxygen and glucose deprivation cell death. J. Pharmacol. Exp. Ther. 323, 70–77 10.1124/jpet.106.119024 17620457

[B16] Cervetto C. Frattaroli D. Maura G. Marcoli M. (2013). Motor neuron dysfunction in a mouse model of ALS: gender-dependent effect of P2X7 antagonism. Toxicology 311, 69–77 10.1016/j.tox.2013.04.004 23583883

[B17] Chakfe Y. Seguin R. Antel J. P. Morissette C. Malo D. Henderson D. (2002). ADP and AMP induce interleukin-1beta release from microglial cells through activation of ATP-primed P2X7 receptor channels. J. Neurosci. 22, 3061–3069 11943809 10.1523/JNEUROSCI.22-08-03061.2002 PMC6757521

[B18] Chen J. W. Wasterlain C. G. (2006). Status epilepticus: pathophysiology and management in adults. Lancet Neurol. 5, 246–256 10.1016/S1474-4422(06)70374-X 16488380

[B19] Chen S. Ma Q. Krafft P. R. Chen Y. Tang J. Zhang J. (2013). P2X7 receptor antagonism inhibits p38 mitogen-activated protein kinase activation and ameliorates neuronal apoptosis after subarachnoid hemorrhage in rats. Crit. Care Med. [Epub ahead of print]. 10.1097/CCM.0b013e31829a8246 23963136 PMC3841260

[B20] Chessell I. P. Simon J. Hibell A. D. Michel A. D. Barnard E. A. Humphrey P. P. (1998). Cloning and functional characterisation of the mouse P2X7 receptor. FEBS Lett. 439, 26–30 10.1016/S0014-5793(98)01332-5 9849870

[B21] Choi H. K. Ryu H. J. Kim J. E. Jo S. M. Choi H. C. Song H. K. (2012). The roles of P2X7 receptor in regional-specific microglial responses in the rat brain following status epilepticus. Neurol. Sci. 33, 515–525 10.1007/s10072-011-0740-z 21845474

[B22] Collo G. Neidhart S. Kawashima E. Kosco-Vilbois M. North R. A. Buell G. (1997). Tissue distribution of the P2X7 receptor. Neuropharmacology 36, 1277–1283 10.1016/S0028-3908(97)00140-8 9364482

[B22a] Collo G. North R. A. Kawashima E. Merlo-Pich E. Neidhart S. Surprenant A. (1996). Cloning of P2X5 and P2X6 receptors and the distribution and properties of an extended family of ATP-gated ion channels. J. Neurosci. 16, 2495–2507 8786426 10.1523/JNEUROSCI.16-08-02495.1996 PMC6578782

[B23] Craigie E. Birch R. E. Unwin R. J. Wildman S. S. (2013). The relationship between P2X4 and P2X7: a physiologically important interaction? Front. Physiol. 4:216 10.3389/fphys.2013.00216 23966951 PMC3744038

[B24] Curia G. Longo D. Biagini G. Jones R. S. Avoli M. (2008). The pilocarpine model of temporal lobe epilepsy. J. Neurosci. Methods 172, 143–157 10.1016/j.jneumeth.2008.04.019 18550176 PMC2518220

[B25] Dale N. Frenguelli B. G. (2009). Release of adenosine and ATP during ischemia and epilepsy. Curr. Neuropharmacol. 7, 160–179 10.2174/157015909789152146 20190959 PMC2769001

[B26] Deuchars S. A. Atkinson L. Brooke R. E. Musa H. Milligan C. J. Batten T. F. (2001). Neuronal P2X7 receptors are targeted to presynaptic terminals in the central and peripheral nervous systems. J. Neurosci. 21, 7143–7152 11549725 10.1523/JNEUROSCI.21-18-07143.2001 PMC6762981

[B27] Devinsky O. Vezzani A. Najjar S. De Lanerolle N. C. Rogawski M. A. (2013). Glia and epilepsy: excitability and inflammation. Trends Neurosci. 36, 174–184 10.1016/j.tins.2012.11.008 23298414

[B28] Diaz-Hernandez J. I. Gomez-Villafuertes R. Leon-Otegui M. Hontecillas-Prieto L. Del Puerto A. Trejo J. L. (2012). *In vivo* P2X7 inhibition reduces amyloid plaques in Alzheimer's disease through GSK3beta and secretases. Neurobiol. Aging 33, 1816–1828 10.1016/j.neurobiolaging.2011.09.040 22048123

[B29] Diaz-Hernandez M. Del Puerto A. Diaz-Hernandez J. I. Diez-Zaera M. Lucas J. J. Garrido J. J. (2008). Inhibition of the ATP-gated P2X7 receptor promotes axonal growth and branching in cultured hippocampal neurons. J. Cell. Sci. 121, 3717–3728 10.1242/jcs.034082 18987356

[B30] Diaz-Hernandez M. Diez-Zaera M. Sanchez-Nogueiro J. Gomez-Villafuertes R. Canals J. M. Alberch J. (2009). Altered P2X7-receptor level and function in mouse models of Huntington's disease and therapeutic efficacy of antagonist administration. FASEB J. 23, 1893–1906 10.1096/fj.08-122275 19171786

[B31] Di Virgilio F. Chiozzi P. Falzoni S. Ferrari D. Sanz J. M. Venketaraman V. (1998). Cytolytic P2X purinoceptors. Cell Death Differ. 5, 191–199 10.1038/sj.cdd.4400341 10200464

[B32] Dona F. Ulrich H. Persike D. S. Conceicao I. M. Blini J. P. Cavalheiro E. A. (2009). Alteration of purinergic P2X4 and P2X7 receptor expression in rats with temporal-lobe epilepsy induced by pilocarpine. Epilepsy Res. 83, 157–167 10.1016/j.eplepsyres.2008.10.008 19084381

[B33] Duan S. Anderson C. M. Keung E. C. Chen Y. Swanson R. A. (2003). P2X7 receptor-mediated release of excitatory amino acids from astrocytes. J. Neurosci. 23, 1320–1328 12598620 10.1523/JNEUROSCI.23-04-01320.2003 PMC6742264

[B34] Engel T. Gomez-Villafuertes R. Tanaka K. Mesuret G. Sanz-Rodriguez A. Garcia-Huerta P. (2012a). Seizure suppression and neuroprotection by targeting the purinergic P2X7 receptor during status epilepticus in mice. FASEB J. 26, 1616–1628 10.1096/fj.11-196089 22198387

[B35] Engel T. Jimenez-Pacheco A. Miras-Portugal M. T. Diaz-Hernandez M. Henshall D. C. (2012b). P2X7 receptor in epilepsy; role in pathophysiology and potential targeting for seizure control. Int. J. Physiol. Pathophysiol. Pharmacol. 4, 174–187 23320131 PMC3544219

[B36] Fabene P. F. Merigo F. Galie M. Benati D. Bernardi P. Farace P. (2007). Pilocarpine-induced status epilepticus in rats involves ischemic and excitotoxic mechanisms. PLoS ONE 2:e1105 10.1371/journal.pone.0001105 17971868 PMC2040510

[B37] Ferrari D. Villalba M. Chiozzi P. Falzoni S. Ricciardi-Castagnoli P. Di Virgilio F. (1996). Mouse microglial cells express a plasma membrane pore gated by extracellular ATP. J. Immunol. 156, 1531–1539 8568257

[B38] Gever J. R. Cockayne D. A. Dillon M. P. Burnstock G. Ford A. P. (2006). Pharmacology of P2X channels. Pflugers Arch. 452, 513–537 10.1007/s00424-006-0070-9 16649055

[B38a] Guo W. Xu X. Gao X. Burnstock G. He C. Xiang Z. (2008). Expression of P2X5 receptors in the mouse CNS. Neuroscience 156, 673–692 10.1016/j.neuroscience.2008.07.062 18773945

[B39] Houser C. R. Zhang N. Peng Z. Huang C. S. Cetina Y. (2012). Neuroanatomical clues to altered neuronal activity in epilepsy: from ultrastructure to signaling pathways of dentate granule cells. Epilepsia 53(Suppl. 1), 67–77 10.1111/j.1528-1167.2012.03477.x 22612811 PMC4214139

[B40] Iglesias R. Locovei S. Roque A. Alberto A. P. Dahl G. Spray D. C. (2008). P2X7 receptor-Pannexin1 complex: pharmacology and signaling. Am. J. Physiol. Cell Physiol. 295, C752–C760 10.1152/ajpcell.00228.2008 18596211 PMC2544446

[B41] Jimenez-Pacheco A. Mesuret G. Sanz-Rodriguez A. Tanaka K. Mooney C. Conroy R. (2013). Increased neocortical expression of the P2X7 receptor after status epilepticus and anticonvulsant effect of P2X7 receptor antagonist A-438079. Epilepsia 54, 1551–1561 10.1111/epi.12257 23808395

[B42] Kang T. C. An S. J. Park S. K. Hwang I. K. Won M. H. (2003). P2X2 and P2X4 receptor expression is regulated by a GABA(A) receptor-mediated mechanism in the gerbil hippocampus. Brain Res. Mol. Brain Res. 116, 168–175 10.1016/S0169-328X(03)00260-2 12941474

[B42a] Kanjhan R. Housley G. D. Burton L. D. Christie D. L. Kippenberger A. Thorne P. R. (1999). Distribution of the P2X2 receptor subunit of the ATP-gated ion channels in the rat central nervous system. J. Comp. Neurol. 407, 11–32 10.1002/(SICI)1096-9861(19990428)407:1<11::AID-CNE2>3.0.CO;2-R 10213185

[B43] Khakh B. S. (2001). Molecular physiology of P2X receptors and ATP signalling at synapses. Nat. Rev. Neurosci. 2, 165–174 10.1038/35058521 11256077

[B44] Khakh B. S. Gittermann D. Cockayne D. A. Jones A. (2003). ATP modulation of excitatory synapses onto interneurons. J. Neurosci. 23, 7426–7437 12917379 10.1523/JNEUROSCI.23-19-07426.2003 PMC6740451

[B45] Khakh B. S. North R. A. (2006). P2X receptors as cell-surface ATP sensors in health and disease. Nature 442, 527–532 10.1038/nature04886 16885977

[B46] Kim J. E. Kang T. C. (2011). The P2X7 receptor-pannexin-1 complex decreases muscarinic acetylcholine receptor-mediated seizure susceptibility in mice. J. Clin. Invest. 121, 2037–2047 10.1172/JCI44818 21505260 PMC3083785

[B47] Kim J. E. Kwak S. E. Jo S. M. Kang T. C. (2009). Blockade of P2X receptor prevents astroglial death in the dentate gyrus following pilocarpine-induced status epilepticus. Neurol. Res. 31, 982–988 10.1179/174313209X389811 19138473

[B48] Kim J. E. Ryu H. J. Yeo S. I. Kang T. C. (2011a). P2X7 receptor differentially modulates astroglial apoptosis and clasmatodendrosis in the rat brain following status epilepticus. Hippocampus 21, 1318–1333 10.1002/hipo.20850 20848604

[B47a] Kim J. E. Ryu H. J. Kang T. C. (2011b). P2X7 receptor activation ameliorates CA3 neuronal damage via a tumor necrosis factor-alpha-mediated pathway in the rat hippocampus following status epilepticus. J. Neuroinflammation 8:62 10.1186/1742-2094-8-62 21631954 PMC3123566

[B49] Kim M. Jiang L. H. Wilson H. L. North R. A. Surprenant A. (2001). Proteomic and functional evidence for a P2X7 receptor signalling complex. EMBO J. 20, 6347–6358 10.1093/emboj/20.22.6347 11707406 PMC125721

[B50] Klaft Z. J. Schulz S. B. Maslarova A. Gabriel S. Heinemann U. Gerevich Z. (2012). Extracellular ATP differentially affects epileptiform activity via purinergic P2X7 and adenosine A1 receptors in naive and chronic epileptic rats. Epilepsia 53, 1978–1986 10.1111/j.1528-1167.2012.03724.x 23106524

[B50a] Lalo U. Pankratov Y. Wichert S. P. Rossner M. J. North R. A. Kirchhoff F. (2008). P2X1 and P2X5 subunits form the functional P2X receptor in mouse cortical astrocytes. J. Neurosci. 28, 5473–5480 10.1523/JNEUROSCI.1149-08.2008 18495881 PMC3844808

[B51] Lazarowski E. R. Boucher R. C. Harden T. K. (2003). Mechanisms of release of nucleotides and integration of their action as P2X- and P2Y-receptor activating molecules. Mol. Pharmacol. 64, 785–795 10.1124/mol.64.4.785 14500734

[B52] Le Feuvre R. A. Brough D. Touzani O. Rothwell N. J. (2003). Role of P2X7 receptors in ischemic and excitotoxic brain injury *in vivo*. J. Cereb. Blood Flow Metab. 23, 381–384 10.1097/00004647-200303000-00013 12621313

[B52a] Le K. T. Villeneuve P. Ramjaun A. R. Mcpherson P. S. Beaudet A. Seguela P. (1998). Sensory presynaptic and widespread somatodendritic immunolocalization of central ionotropic P2X ATP receptors. Neuroscience 83, 177–190 10.1016/80306-4522(97)00365-5 9466408

[B53] Li T. Ren G. Lusardi T. Wilz A. Lan J. Q. Iwasato T. (2008). Adenosine kinase is a target for the prediction and prevention of epileptogenesis in mice. J. Clin. Invest. 118, 571–582 10.1172/JCI33737 18172552 PMC2157568

[B54] Liu G. Gu B. He X. P. Joshi R. B. Wackerle H. D. Rodriguiz R. M. (2013). Transient inhibition of TrkB kinase after status epilepticus prevents development of temporal lobe epilepsy. Neuron 79, 31–38 10.1016/j.neuron.2013.04.027 23790754 PMC3744583

[B55] Lopatar J. Dale N. Frenguelli B. G. (2011). Minor contribution of ATP P2 receptors to electrically-evoked electrographic seizure activity in hippocampal slices: evidence from purine biosensors and P2 receptor agonists and antagonists. Neuropharmacology 61, 25–34 10.1016/j.neuropharm.2011.02.011 21338615

[B56] Loscher W. (2002). Animal models of epilepsy for the development of antiepileptogenic and disease-modifying drugs. A comparison of the pharmacology of kindling and post-status epilepticus models of temporal lobe epilepsy. Epilepsy Res. 50, 105–123 10.1016/S0920-1211(02)00073-6 12151122

[B57] Loscher W. (2009). Molecular mechanisms of drug resistance in status epilepticus. Epilepsia 50(Suppl. 12), 19–21 10.1111/j.1528-1167.2009.02367.x 19941514

[B58] Lowenstein D. H. (2005). Treatment options for status epilepticus. Curr. Opin. Pharmacol. 5, 334–339 10.1016/j.coph.2005.04.003 15907922

[B59] Marcellino D. Suarez-Boomgaard D. Sanchez-Reina M. D. Aguirre J. A. Yoshitake T. Yoshitake S. (2010). On the role of P2X(7) receptors in dopamine nerve cell degeneration in a rat model of Parkinson's disease: studies with the P2X(7) receptor antagonist A-438079. J. Neural Transm. 117, 681–687 10.1007/s00702-010-0400-0 20387084

[B60] Marchi N. Fan Q. Ghosh C. Fazio V. Bertolini F. Betto G. (2009). Antagonism of peripheral inflammation reduces the severity of status epilepticus. Neurobiol. Dis. 33, 171–181 10.1016/j.nbd.2008.10.002 19010416 PMC3045783

[B61] Masin M. Young C. Lim K. Barnes S. J. Xu X. J. Marschall V. (2012). Expression, assembly and function of novel C-terminal truncated variants of the mouse P2X7 receptor: re-evaluation of P2X7 knockouts. Br. J. Pharmacol. 165, 978–993 10.1111/j.1476-5381.2011.01624.x 21838754 PMC3312493

[B62] Matute C. Torre I. Perez-Cerda F. Perez-Samartin A. Alberdi E. Etxebarria E. (2007). P2X(7) receptor blockade prevents ATP excitotoxicity in oligodendrocytes and ameliorates experimental autoimmune encephalomyelitis. J. Neurosci. 27, 9525–9533 10.1523/JNEUROSCI.0579-07.2007 17728465 PMC6673129

[B63] McGaraughty S. Chu K. L. Namovic M. T. Donnelly-Roberts D. L. Harris R. R. Zhang X. F. (2007). P2X7-related modulation of pathological nociception in rats. Neuroscience 146, 1817–1828 10.1016/j.neuroscience.2007.03.035 17478048

[B64] Melani A. Amadio S. Gianfriddo M. Vannucchi M. G. Volonte C. Bernardi G. (2006). P2X7 receptor modulation on microglial cells and reduction of brain infarct caused by middle cerebral artery occlusion in rat. J. Cereb. Blood Flow Metab. 26, 974–982 10.1038/sj.jcbfm.9600250 16395292

[B65] Monif M. Reid C. A. Powell K. L. Smart M. L. Williams D. A. (2009). The P2X7 receptor drives microglial activation and proliferation: a trophic role for P2X7R pore. J. Neurosci. 29, 3781–3791 10.1523/JNEUROSCI.5512-08.2009 19321774 PMC6665035

[B66] Mouri G. Jimenez-Mateos E. Engel T. Dunleavy M. Hatazaki S. Paucard A. (2008). Unilateral hippocampal CA3-predominant damage and short latency epileptogenesis after intra-amygdala microinjection of kainic acid in mice. Brain Res. 1213, 140–151 10.1016/j.brainres.2008.03.061 18455706

[B67] Murphy N. Cowley T. R. Richardson J. C. Virley D. Upton N. Walter D. (2012). The neuroprotective effect of a specific P2X(7) receptor antagonist derives from its ability to inhibit assembly of the NLRP3 inflammasome in glial cells. Brain Pathol. 22, 295–306 10.1111/j.1750-3639.2011.00531.x 21933296 PMC8092963

[B68] Nicke A. Kuan Y. H. Masin M. Rettinger J. Marquez-Klaka B. Bender O. (2009). A functional P2X7 splice variant with an alternative transmembrane domain 1 escapes gene inactivation in P2X7 knock-out mice. J. Biol. Chem. 284, 25813–25822 10.1074/jbc.M109.033134 19546214 PMC2757983

[B69] Norenberg W. Illes P. (2000). Neuronal P2X receptors: localisation and functional properties. Naunyn Schmiedebergs Arch. Pharmacol. 362, 324–339 10.1007/s002100000311 11111827

[B70] North R. A. (2002). Molecular physiology of P2X receptors. Physiol. Rev. 82, 1013–1067 12270951 10.1152/physrev.00015.2002

[B71] North R. A. Jarvis M. F. (2013). P2X receptors as drug targets. Mol. Pharmacol. 83, 759–769 10.1124/mol.112.083758 23253448 PMC3608433

[B72] North R. A. Surprenant A. (2000). Pharmacology of cloned P2X receptors. Annu. Rev. Pharmacol. Toxicol. 40, 563–580 10.1146/annurev.pharmtox.40.1.563 10836147

[B73] Pankratov Y. V. Lalo U. V. Krishtal O. A. (2002). Role for P2X receptors in long-term potentiation. J. Neurosci. 22, 8363–8369 12351710 10.1523/JNEUROSCI.22-19-08363.2002 PMC6757784

[B74] Papp L. Balazsa T. Kofalvi A. Erdelyi F. Szabo G. Vizi E. S. (2004). P2X receptor activation elicits transporter-mediated noradrenaline release from rat hippocampal slices. J. Pharmacol. Exp. Ther. 310, 973–980 10.1124/jpet.104.066712 15084650

[B75] Pelegrin P. Surprenant A. (2006). Pannexin-1 mediates large pore formation and interleukin-1beta release by the ATP-gated P2X7 receptor. EMBO J. 25, 5071–5082 10.1038/sj.emboj.7601378 17036048 PMC1630421

[B76] Peng W. Cotrina M. L. Han X. Yu H. Bekar L. Blum L. (2009). Systemic administration of an antagonist of the ATP-sensitive receptor P2X7 improves recovery after spinal cord injury. Proc. Natl. Acad. Sci. U.S.A. 106, 12489–12493 10.1073/pnas.0902531106 19666625 PMC2718350

[B77] Rappold P. M. Lynd-Balta E. Joseph S. A. (2006). P2X7 receptor immunoreactive profile confined to resting and activated microglia in the epileptic brain. Brain Res. 1089, 171–178 10.1016/j.brainres.2006.03.040 16635480

[B78] Ross F. M. Brodie M. J. Stone T. W. (1998). Modulation by adenine nucleotides of epileptiform activity in the CA3 region of rat hippocampal slices. Br. J. Pharmacol. 123, 71–80 10.1038/sj.bjp.0701586 9484856 PMC1565143

[B78a] Rubio M. E. Soto F. (2001). Distinct Localization of P2X receptors at excitatory postsynaptic specializations. J. Neurosci. 21, 641–653 11160443 10.1523/JNEUROSCI.21-02-00641.2001 PMC6763822

[B78b] Seguela P. Haghighi A. Soghomonian J. J. Cooper E. (1996). A novel neuronal P2x ATP receptor ion channel with widespread distribution in the brain. J. Neurosci. 16, 448–455 8551329 10.1523/JNEUROSCI.16-02-00448.1996 PMC6578647

[B79] Shorvon S. (2011). The treatment of status epilepticus. Curr. Opin. Neurol. 24, 165–170 10.1097/WCO.0b013e3283446f31 21378567

[B80] Sim J. A. Young M. T. Sung H. Y. North R. A. Surprenant A. (2004). Reanalysis of P2X7 receptor expression in rodent brain. J. Neurosci. 24, 6307–6314 10.1523/JNEUROSCI.1469-04.2004 15254086 PMC6729549

[B81] Skaper S. D. Debetto P. Giusti P. (2010). The P2X7 purinergic receptor: from physiology to neurological disorders. FASEB J. 24, 337–345 10.1096/fj.09-138883 19812374

[B82] Slaughter L. A. Patel A. D. Slaughter J. L. (2013). Pharmacological treatment of neonatal seizures: a systematic review. J. Child Neurol. 28, 351–364 10.1177/0883073812470734 23318696 PMC3805825

[B83] Solle M. Labasi J. Perregaux D. G. Stam E. Petrushova N. Koller B. H. (2001). Altered cytokine production in mice lacking P2X(7) receptors. J. Biol. Chem. 276, 125–132 10.1074/jbc.M006781200 11016935

[B84] Sperk G. (1994). Kainic acid seizures in the rat. Prog. Neurobiol. 42, 1–32 10.1016/0301-0082(94)90019-1 7480784

[B85] Sperlagh B. Heinrich A. Csolle C. (2007). P2 receptor-mediated modulation of neurotransmitter release-an update. Purinergic Signal. 3, 269–284 10.1007/s11302-007-9080-0 18404441 PMC2072919

[B86] Surprenant A. Rassendren F. Kawashima E. North R. A. Buell G. (1996). The cytolytic P2Z receptor for extracellular ATP identified as a P2X receptor (P2X7). Science 272, 735–738 10.1126/science.272.5262.735 8614837

[B87] Synowiec A. S. Singh D. S. Yenugadhati V. Valeriano J. P. Schramke C. J. Kelly K. M. (2013). Ketamine use in the treatment of refractory status epilepticus. Epilepsy Res. 105, 183–188 10.1016/j.eplepsyres.2013.01.007 23369676

[B88] Trang T. Beggs S. Salter M. W. (2012). ATP receptors gate microglia signaling in neuropathic pain. Exp. Neurol. 234, 354–361 10.1016/j.expneurol.2011.11.012 22116040 PMC3748033

[B89] Ulmann L. Levavasseur F. Avignone E. Peyroutou R. Hirbec H. Audinat E. (2013). Involvement of P2X4 receptors in hippocampal microglial activation after status epilepticus. Glia 61, 1306–1319 10.1002/glia.22516 23828736

[B90] Vessey D. A. Li L. Kelley M. (2011). Ischemic preconditioning requires opening of pannexin-1/P2X(7) channels not only during preconditioning but again after index ischemia at full reperfusion. Mol. Cell. Biochem. 351, 77–84 10.1007/s11010-011-0713-9 21267638

[B91] Vezzani A. Balosso S. Maroso M. Zardoni D. Noe F. Ravizza T. (2010). ICE/caspase 1 inhibitors and IL-1beta receptor antagonists as potential therapeutics in epilepsy. Curr. Opin. Investig. Drugs 11, 43–50 20047158

[B92] Virginio C. Mackenzie A. Rassendren F. A. North R. A. Surprenant A. (1999). Pore dilation of neuronal P2X receptor channels. Nat. Neurosci. 2, 315–321 10.1038/7225 10204537

[B93] Wasterlain C. G. Chen J. W. (2006). Definition and classification of status epilepticus, in Status Epilepticus: Mechanisms and Management, eds Wasterlain C. G. Treiman D. M. (Cambridge, MA: MIT Press), 11–16

[B94] Wieraszko A. Seyfried T. N. (1989). Increased amount of extracellular ATP in stimulated hippocampal slices of seizure prone mice. Neurosci. Lett. 106, 287–293 10.1016/0304-3940(89)90178-X 2532311

[B95] Yanagisawa D. Kitamura Y. Takata K. Hide I. Nakata Y. Taniguchi T. (2008). Possible involvement of P2X7 receptor activation in microglial neuroprotection against focal cerebral ischemia in rats. Biol. Pharm. Bull. 31, 1121–1130 10.1248/bpb.31.1121 18520042

[B96] Yu Q. Guo Z. Liu X. Ouyang Q. He C. Burnstock G. (2013). Block of P2X7 receptors could partly reverse the delayed neuronal death in area CA1 of the hippocampus after transient global cerebral ischemia. Purinergic Signal. [Epub ahead of print]. 10.1007/s11302-013-9379-y 23877788 PMC3889395

[B97] Yu Y. Ugawa S. Ueda T. Ishida Y. Inoue K. Kyaw Nyunt A. (2008). Cellular localization of P2X7 receptor mRNA in the rat brain. Brain Res. 1194, 45–55 10.1016/j.brainres.2007.11.064 18177631

[B98] Ziemann A. E. Schnizler M. K. Albert G. W. Severson M. A. Howard M. A. 3rd. Welsh M. J. (2008). Seizure termination by acidosis depends on ASIC1a. Nat. Neurosci. 11, 816–822 10.1038/nn.2132 18536711 PMC2553357

